# A method for simulating forward falls and controlling impact velocity

**DOI:** 10.1016/j.mex.2023.102399

**Published:** 2023-09-26

**Authors:** James Borrelli, Robert A. Creath, Mark W. Rogers

**Affiliations:** a Biomedical Engineering, Stevenson University; b Department of Exercise Science, Lebanon Valley College; c Department of Physical Therapy and Rehabilitation Science, University of Maryland

**Keywords:** Upper extremity, Forward falls, Fall-injury, Biomechanics, FALL simulator For Injury prevention Training and assessment

## Abstract

Assessment of protective arm reactions associated with forward falls are typically performed by dropping research participants from a height onto a landing surface. The impact velocity is generally modulated by controlling the total height of the fall. This contrasts with an actual fall where the fall velocity is dependent on several factors in addition to fall height and not likely predictable at the onset of the fall. A counterweight and pulley system can be used to modulate the fall velocity in simulated forward falls in a manner that is not predictable to study participants, enhancing experimental validity. However, predicting the fall velocity based on participant height and weight and counterweight mass is not straightforward. In this article, the design of the FALL simulator For Injury prevention Training and assessment (FALL FIT) system is described. A dynamic model of the FALL FIT and counterweight system is developed and model parameters are fit using nonlinear optimization and experimental data. The fitted model enables prediction of fall velocity as a function of participant height and weight and counterweight load. The method can be used to provide controllable perturbations thereby elucidating the control strategy used when protecting the body from injury in a forward fall, how the control strategy changes because of aging or dysfunction or as a method for progressive protective arm reaction training.

• Construction of device to simulate forward falls with controllable impact velocity using material that are commercially available is described
• A dynamic model of the FALL FIT is developed to estimate the impact velocity of a simulated forward fall using participant height and counterweight load
• The dynamic model is validated using data from 3 previous studies

## Graphical abstract

**Figure:**
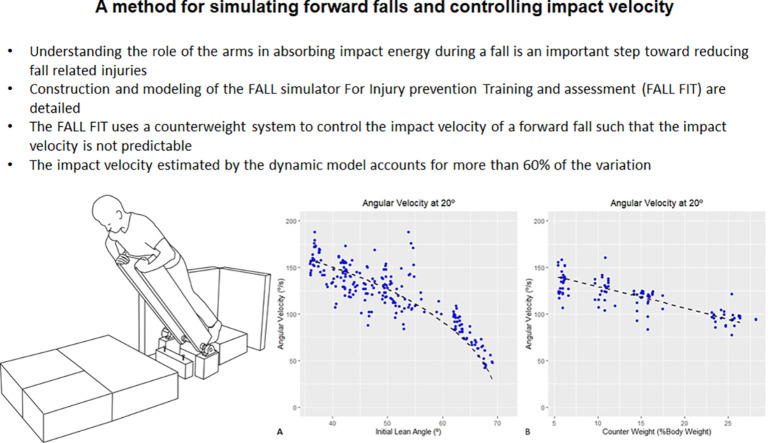

Specifications table

**Table:** 

Subject area	Medicine and Dentistry
More specific subject area:	Rehabilitation
Name of your method:	FALL simulator For Injury prevention Training and assessment
Name and reference of original method:	n/a
Resource availability:	n/a

**Method details**

Falls are a leading cause of hip fractures, traumatic brain injuries, and wrist fractures [1], [2], [3]. Forward falls are among the most common fall direction among older adults [4], [5], [6], [7]. While lateral falls may be more often associated with hip fracture, the orientation at impact is often different that the initial fall direction [7]. For example, forward falls have been associated with increased risk of hip fracture in residents in long-term care [8]. Protective arm reactions in response to a fall are thought to reduce the likelihood of injury [9], [10], [11], [12], [13]. However, these efforts appear to decline with age [7,8,14] and falls result in more frequent injuries and death in adults over 65 years old [15].

Protective arm reactions require a rapid reaction and orientation of the body and arms in preparation for impact, following a loss of balance, and subsequent controlled deceleration post-impact [16,17]. Several different experimental methods have been developed [10,11,[18], [19], [20], [21], [22]] in an effort to enhance our understanding of effective protective reactions in falls. A consistent finding has been that protective arm reactions are affected by sex and age [23], [24], [25], [26], [27], [28], [29].

Other methods of investigating falls involve simulating realistic fall conditions,observing actual falls using a translating platform, or using surveillance systems respectively [7,10,11,14,30]. These methods have provided invaluable insight into the biomechanics of falls, the fall dynamics (e.g., fall direction, fall speed, body segment impacting the ground), environmental factors (e.g. availability of handholds, constraints on stepping or grasping reactions), however the evoked stepping and arm reactions often used while attempting to maintain balance results in variable outcomes in the impact characteristics causing difficulties comparing across methods and/or trials. For example, following an initial forward fall, individuals may or may not reach-to-grasp a handhold if one is available, attempt to recover balance, or prepare for impact. The success or effectiveness of these initial reactions will affect the orientation and impact velocity.

Alternatively, there are experimental approaches that constrain the available reaction such that a more uniform response is evoked. While these approaches do allow focused study on a particular set of fall dynamics or evoked protective reactions, the perturbation leading to a fall is generally more predictable and less likely to elicit natural reactive responses to fall provoking circumstances. For example, studies of forward falls do not allow for protective stepping responses and make balance recovery options more limited [18,21,26,27,[31], [32], [33]]. Additionally, the impact velocity, which increases with increasing fall heights, is controlled by limiting the fall height to a pre-determined distance which may allow participants to pre-plan a protective response [18,21,26,27,[31], [32], [33]]. Preplanning may result in improved performance in experiments requiring rapid postural responses and may obscure age-related changes [34] or other deterioration of protective biomechanics.

To further address these methodological issues, we have developed a novel perturbation system to simulate forward falls and provides a means to control fall acceleration and velocity using a counterweight system. The design and construction of the device is detailed, a dynamic model of the device is developed, and experimental results are described and used to establish a relationship between model parameters and participant height. Although the methods employed are specific to the FALL FIT, the counterweight system could be adapted to augment other experimental protocols.

## Device description

**Fig. 1:**
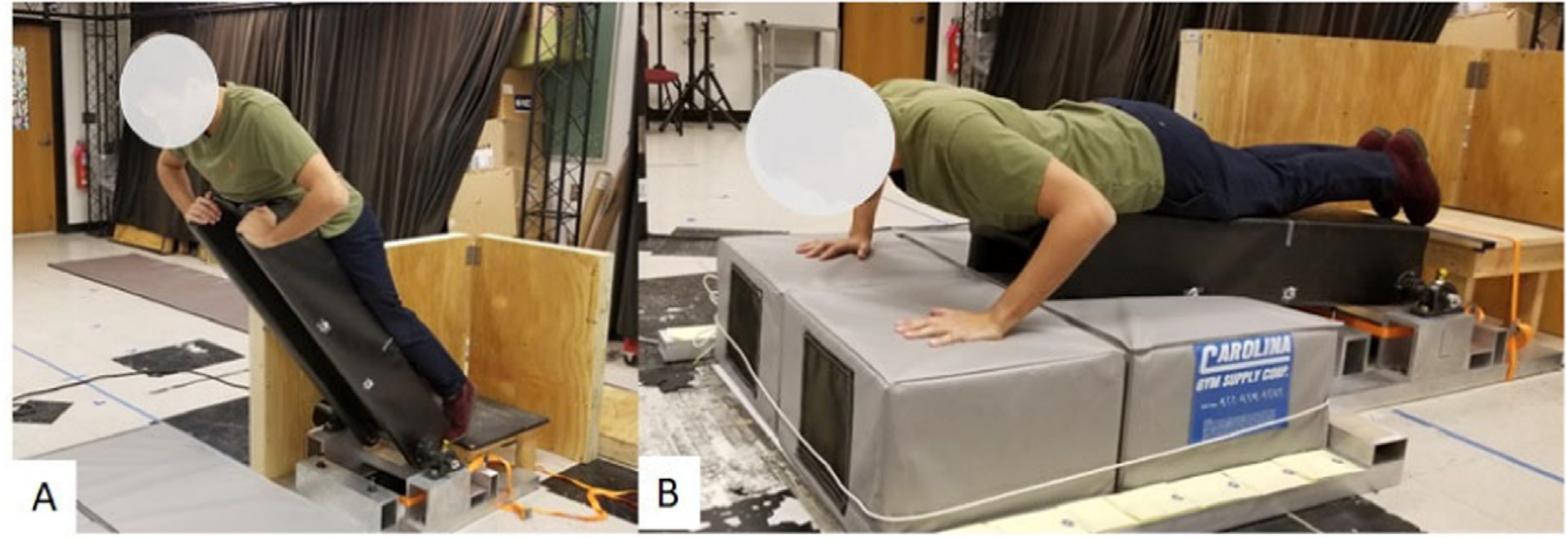
FALL simulator For Injury prevention Training and assessment (FALL FIT) system was designed to simulate a forward fall and require rapid arm orientation prior to impact. Participants lean against the support platform and place their hands on the upper portion of the support platform (**A**). At an unpredictable time, the platform is released requiring participants to rapidly orient their hands and arms to absorb the impact of the fall (**B**). A strap (gait belt) is used to secure participants to the support platform (not shown). The gait-belt is placed slightly above the participant's waist.

The FALL simulator For Injury prevention Training and assessment (FALL FIT) system is shown in Fig. 1. The device is configured as an inverted pendulum upon which study participants lay in a prone position. At an unpredictable time, an electromagnet releases the device from an adjustable leaning position (fall height) and the device and participant fall under the acceleration of gravity. The angular velocity at impact can be modulated by altering the initial lean angle and/or altering the counterweight (Fig. 1**D**). The device was designed in Solidworks (Concord, MA) and machining was carried out by Micro-fabrication, Machining and Electronics Technical Service Center (Catonsville, MD). Materials were sourced from McMaster-Carr Supply Company (McMaster-Carr, Elmhurst, IL).

**Fig. 2:**
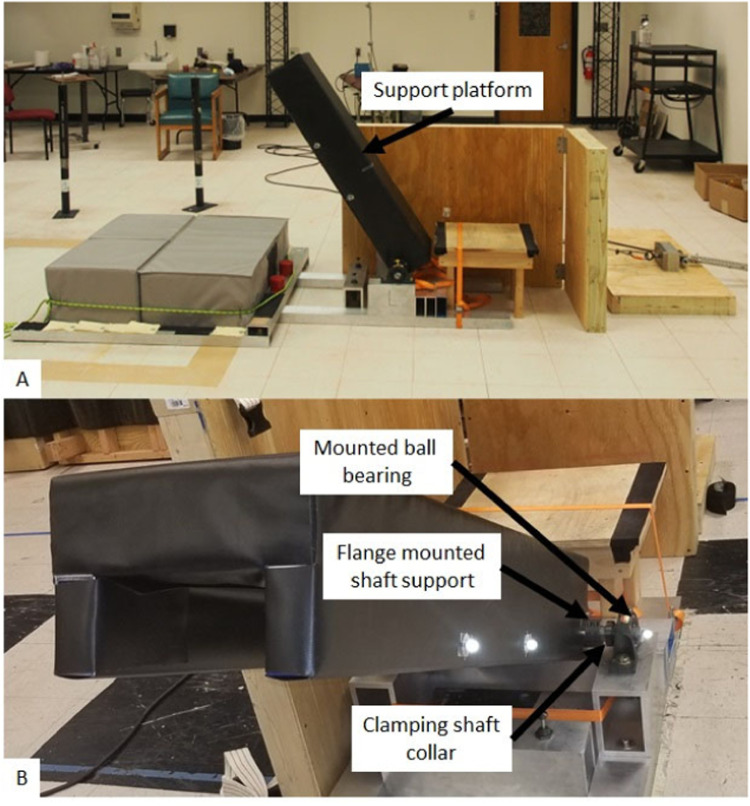
The support platform consists of a pair of parallel rectangular tubes with an aluminum sheet connecting the tubes. The top of the support platform is covered with 12-inch-thick polyester cushions and wrapped with polyester fabric (**A**). The support platform makes an inverted “U” shape when viewed from the front (**B**). A 1-1/4-inch diameter shaft passes longitudinally through the base of the support platform. The shaft is fixed to the support platform with 4 flange mounted shaft supports. The shaft is supported with a pair of mounted ball bearings. Four clamping shaft collars are used to prevent the support platform from shifting laterally on the axle or in the mounted ball bearings.

The support platform that the participants lay on is comprised of two 2” x 4” x 48”-1/8” wall thickness square aluminum tubes. The square tubes are covered with a 12” x 48”-0.1” thick aluminum plate and polyurethane foam cushion (12” x 48”-12” thick), which are all wrapped with polyester fabric (Fig. 2**A**).

The support platform pivots about its most proximal aspect (with respect to the floor). The table pivots about a 1.25” diameter steel axle that is secured to the table using 4 clamping shaft collars. The axle is supported by a pair of mounted ball bearing with cast iron housings which are attached to mounting points in the lab (Fig. 2**B**).

A pair of mounting points were constructed in the lab and consist of 4”x6”x12”-1/2” wall thickness square aluminum (the letter “L” is written on left mounting point shown in Fig. 2) tube stacked on top of a 2”x4”x72”-1/8” wall thickness square aluminum tubes that are bolted to the floor.

**Fig. 3:**
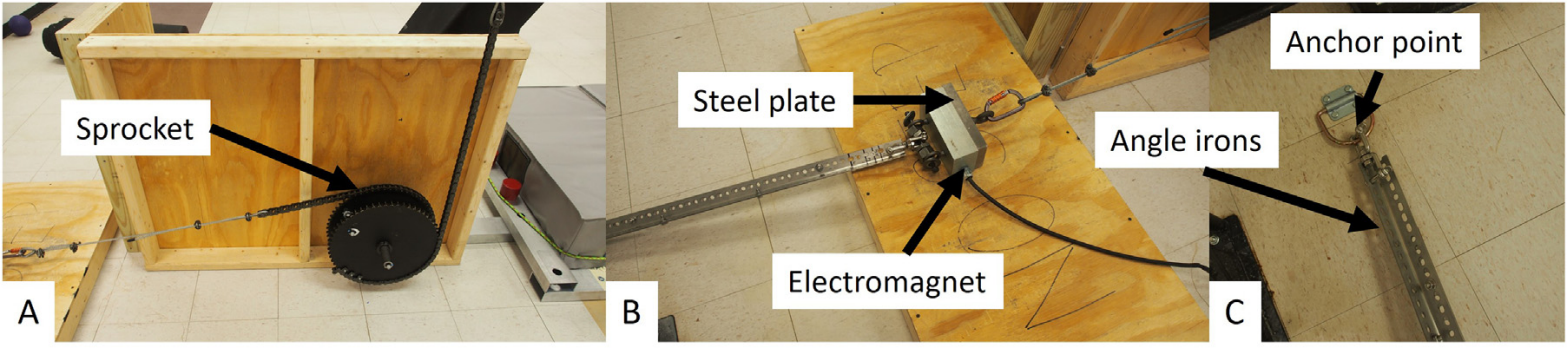
The support platform of the FALL FIT is held in an intial lean angle with a mechanical connection. The shaft of the support platform has a [inner] sprocket and chain that rotate with the support platform (A). The chain is connected to wire rope and then a clevis-to-clevis swivel and an electromagnet (B). On the opposite side of the electromagnet is a another clevis-to-clevis swivel and there are a pair of ‘angle-irons’ that are bolted together to control the initial lean angle (**B**). The angle irons are connected to an anchor point that is built into the laboratory floor (C). The angle of the support platform is controlled by varying the distance between the anchor point and the electromagnetic by increasing or decreasing the length of the pair of angle-irons that are bolted together by altering the among of overlap between the two angle-irons. The outer sprocket is used to raise a counterweight and discussed elsewhere.

The support platform is held in place by an electromagnet (MagneTool Inc., Troy, MI, Fig. 3**A**), which is secured to an anchor point that is bolted to the floor (Fig. 3**B**). The electromagnet is attached to a pair of 1.5”x1.5”x30” steel angle irons that are bolted together. The distance between the anchor and the electromagnet can be controlled by altering the amount of overlap between the two angle-irons resulting in a varying initial lean angle of the support platform of the inverted pendulum. The electromagnet holds a 4”x 8”x1” steel plate that holds the support platform in position. The steel plate is connected to the support platform via a roller chain and 14.76” diameter steel sprocket that is attached to the keyed 1.25” diameter steel axle upon which the table rides (Fig. 3**C**).

**Fig. 4:**
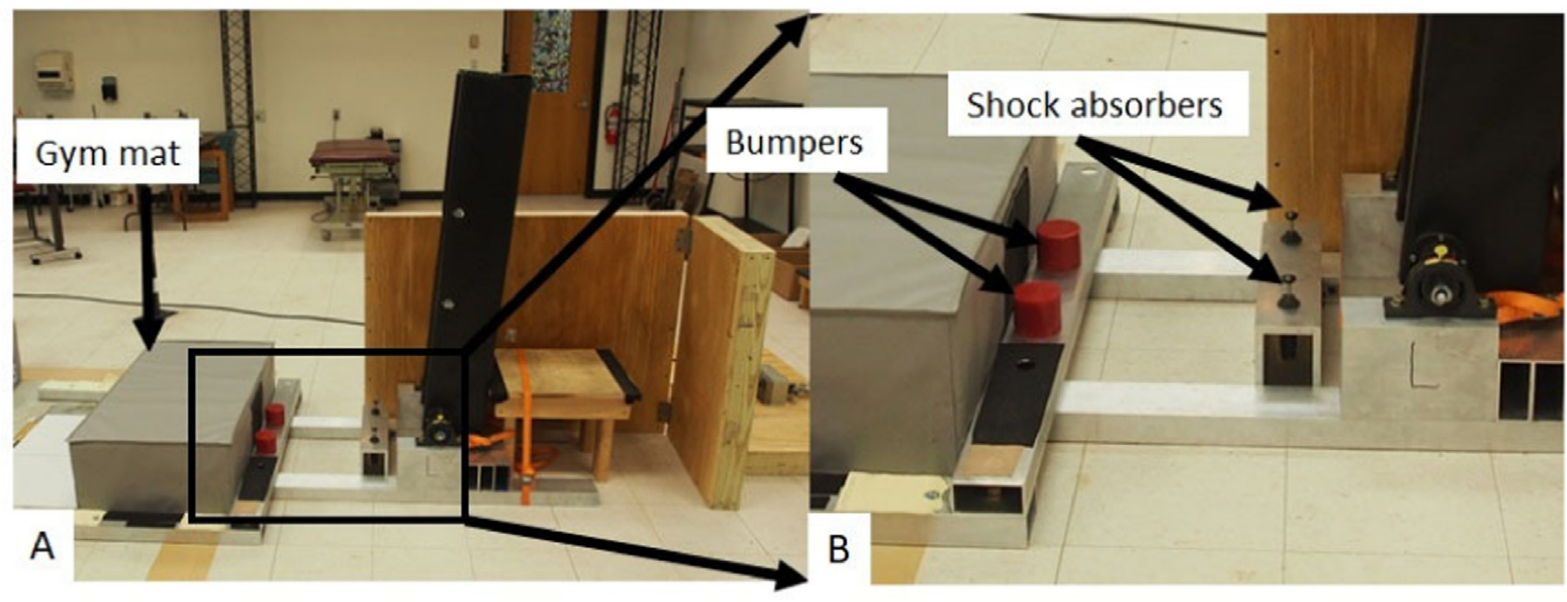
A system of shock absorbers, a gymnastic mat, and bumpers act to assist in absorbing the impact and in trials where the participant is unable to absorb the impact of the fall prior to the support platform reaching horizontal. When the support platform reaches horizontal, it contacts a gymnastic mat (**A**) and a pair of shock absorbers (**B**). After passing 5 degrees below horizontal, the support platform contacts a pair of bumpers.

When the support platform angle reaches about 5 degrees above horizontal, (*θ* = 85 degrees, Fig. 4), the underside of the support platform contacts 12-inch-thick gymnastic mats (Carolina Gym Supply, Hillsborough, NC). When the support platform reaches horizontal (*θ* = 90 degrees), the underside of the platform contacts a pair of 600 in-lb energy capacity shock absorbers and at 5 degrees below the horizontal, the pendulum contacts a pair of heavy-duty polyurethane bumpers and further rotation is prevented.

**Fig. 5:**
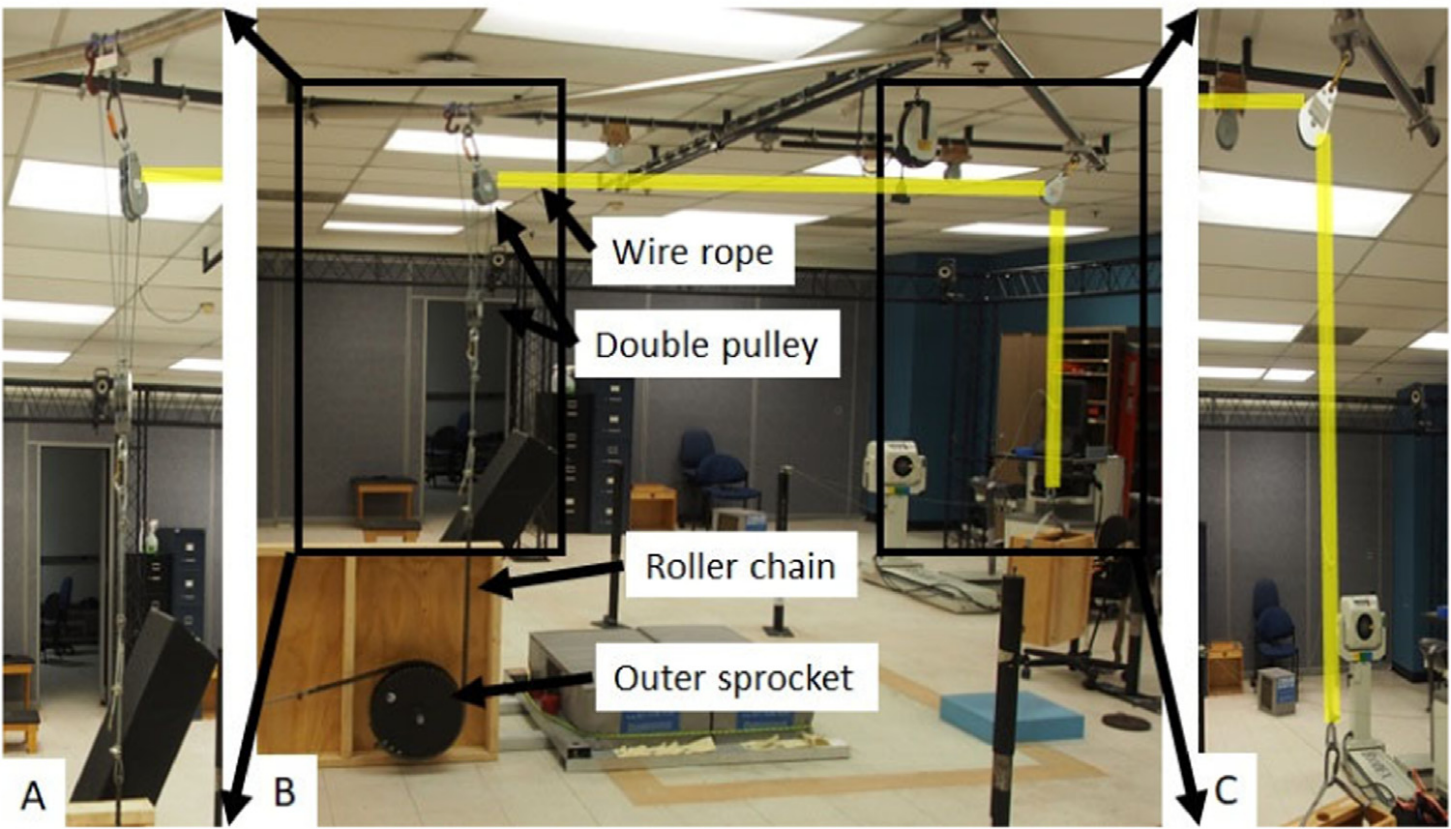
The FALL FIT uses a counterweight to control the fall acceleration and resulting impact velocity. The counterweight is connected to the support platform via a system of pulleys. There is a second, outer, sprocket attached to the axle of the support platform. A roller chain is attached to the outer sprocket and affixed to a double pulley with wire rope (**A**). Wire rope is secured to a post in the ceiling and threaded through 2 double pulleys. The wire rope traverses the ceiling of the lab (highlighted in yellow, **B**) and is threaded through another pulley (**C**) before attaching to a counterweight.

The FALL FITT makes use of a counterweight system similar to the systems that have been used to study drop landings [35], [36], [37], [38]. In studies of drop landings, a participant stands on top of a platform that suddenly lowers. As the platform lowers a counterweight is lifted and derivation of the the governing equations is relatively straight-forward. However, the FALL-FITT requires a more complicated solution. A novel pulley system is required to incorporate a counterweight system with the ‘falling frame’ of the FALL FIT to prevent the cabling of the pulley system from interfering with arm movements. A system of 4 pulleys and a counterweight were used to control angular acceleration and resulting angular velocity. The counterweight system was included by adding an outer sprocket to the axle (Fig. 5**B**). The second sprocket has a roller chain connected to steel rope. The steel rope is threaded through 4 pulleys (Fig. 5**A**) and run across the ceiling and through another pulley where is it attached to a counterweight (Fig. 5**C**).

## Dynamic Model

**Fig. 6:**
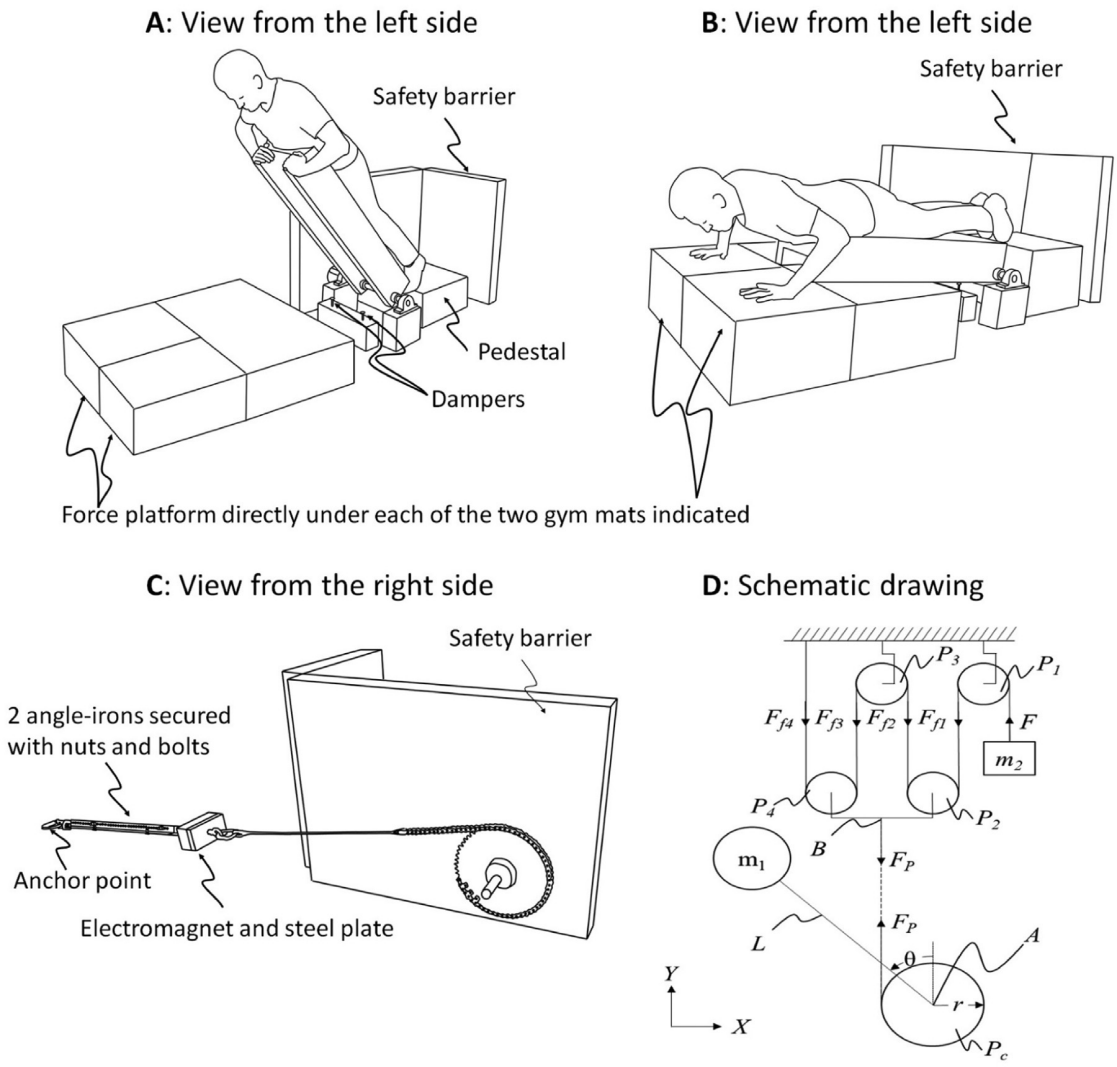
Illustration of the fall simulator. Participants lay on top of the device. At an unpredictable time, the participant is released from a leaning position (panel **A**). Following perturbation onset, the participant rapidly orients their hands and arms to absorb the impact energy (panel **B**). The device is held in an initial leaning position using an electromagnet. The initial lean angle is controlled by varying the distance between the anchor point and the electromagnet (panel **C**). A schematic diagram of the fall simulator with a counterweight system is shown in panel **D**. The participant and support platfrom are modeled as an inverted pendulum with mass *m_1_* and height *L*. When the system is released, the angle *θ* increases, pulling point *B* downward. The counterweight *m_2_* moves upward at a rate that is 4 times greater than point *B* moves downward. Four pulleys, *P_1_, P_2_, P_3_*, and *P_4_*, were used to effectively multiply the pulley/counterweight force *F_p_* due to the counterweight *m_2_*. The pulley system was an effort to compensate for the relatively small moment arm the counterweight force acts through compared to the inverted pendulum length, *r* versus *L* respectively. Physical constraints limited the maximum allowable radius *r* of the counterweight pulley (*P_c_*). The cable system needed to be sufficiently far from the area where the arms could move during the trials. Additionally, pivot point *A* had to be higher above the ground than the radius of the counterweight pulley. Adapted from Borrelli, Creath, Westlake, and Rogers (2023) with permission [39].

A schematic of the system is shown in Fig. 6. The participant and support platform are idealized as an inverted pendulum with mass *m_1_* located a distance *L* from the axis of rotation at point *A*. The distance from the axis of rotation at point A is presumed to be the height of the center of mass (CoM) of the participant and support platform. When the pendulum is released, the pendulum angle *θ* increases. Varying the counterweight provides a means of modulating the angular velocity. Summing the moments about point *A* of the pendulum (Fig. 6**D**) gives

(1) $$I\ddot{\theta }=m_{1}gL\;\sin\theta -F_{P}r.$$

where *I* is the mass moment of inertia of the pendulum, *m_1_* is the mass of the pendulum, *g* is the acceleration due to gravity, *L* is the moment arm of the pendulum or the CoM height, *θ* is the angle of the pendulum, *r* is the radius of the counterweight pulley (*P_c_*), and *F_P_* is the tension in the cable between the *P_c_* and point *B*.

For the counterweight and pully system, we sum the forces in the *Y*-direction of the counterweight

(2) $$F-m_{2}g=m_{2}a_{y}$$

where *m_2_* is the mass of the counterweight, *F* is the tension in the cable supporting *m_2_*, and *a_y_* is the vertical acceleration of the counterweight (*m_2_*). The displacement of point *B* is a function of the product of the change in angle *θ* and the radius of the gear

(3) ΔB=Δθr.

It can be shown that the vertical displacement of *m_2_* (y_m2_) is 4 times greater than the displacement of point *B*, therefore

(4) $$y_{m_{2}}=4\Delta \theta r.$$

Differentiating eqn [4] twice yields a relationship between the vertical acceleration of the counterweight (a_y_) and the angle of the inverted pendulum

(5) $$a_{y}=4r\ddot{\theta }.$$

Substituting eqn [5] into eqn [2] and rearranging yields a function for the tension, *F*, in the cable above the counterweight

(6) $$F=4m_{2}r\ddot{\theta }+m_{2}g.$$

The tension in the cable between pulley 1 (*P_1_*) and pulley 2 (*P_2_*) is governed by the Euler-Eytelwein, or capstan, equation (see Lubarda, 2014 for a summary [40]). The tension in the cable between *P_1_* and *P_2_* is a function of the drag friction, *μ_F_*,

(7) $$F_{f1}=Fe^{\mu_{F}\pi }=\left(4m_{2}r\ddot{\theta }+m_{2}\right)e^{\mu_{F}\pi }.$$

The tension in the cable between pulley 2 (*P_2_*) and pulley 3 (*P_3_*) is then

(8) $$F_{f2}=F_{f1}e^{\mu_{F}\pi }=\left(\left(4m_{2}r\ddot{\theta }+m_{2}g\right)e^{\mu_{F}\pi }\right)e^{\mu_{F}\pi }=\left(4m_{2}r\ddot{\theta }+m_{2}g\right)e^{2\mu_{F}\pi }.$$

It can be shown that

(9) $$F_{P}=F_{f1}+F_{f2}+F_{f3}+F_{f4}=\left(4m_{2}r\ddot{\theta }+m_{2}g\right)\left(e^{\mu_{F}\pi }+e^{2\mu_{F}\pi }+e^{3\mu_{F}\pi }+e^{4\mu_{F}\pi }\right).$$

Substituting eqn [9] into eqn [1] gives

(10) $$I\ddot{\theta }=\;m_{1}gL\;\sin\theta -\left(4m_{2}r^{2}\ddot{\theta }+m_{2}rg\right)\left(e^{\mu_{F}\pi }+e^{2\mu_{F}\pi }+e^{3\mu_{F}\pi }+e^{4\mu_{F}\pi }\right).$$

For convenience let

(11) $$C_{1}=\left(e^{\mu_{F}\pi }+e^{2\mu_{F}\pi }+e^{3\mu_{F}\pi }+e^{4\mu_{F}\pi }\right).$$

Rearranging eqn [10] gives

(12) $$\ddot{\theta }=\frac{m_{1}gL\;\sin\theta -C_{1}m_{2}rg}{I+4C_{1}m_{2}r^{2}}.$$

Dividing the numerator and denominator of eqn [12] by *m_1_* gives a function for the angular acceleration in terms of the counterweight ratio (*m_2_*/*m_1_*)

(13) $$\ddot{\theta }=\frac{gL\;\sin\theta -C_{1}rgm_{2}/m_{1}}{L^{2}+4C_{1}r^{2}m_{2}/m_{1}}.$$

In the case that the counterweight is equal to zero, eqn [13] reduces to the characteristic equation of an inverted pendulum

(14) $$\ddot{\theta }=\frac{g}{L}\;\sin\theta .$$

## Protocol

The data from two previous experiments was used to parameterize the effect of initial lean angle and counterweight load on impact velocity. The aim of these previous studies was to enhance our understanding of the effect of age [[18], [25]] and impact velocity [39] on the biomechanics of protective arm reactions during forward falls. The results from these studies are reported elsewhere [[18], [25],^39^]. The data from the experiment concerned with the effect of age was used to parameterize the effect of initial lean angle on impact velocity. In this experiment, the initial lean angle was varied with no counterweight. The data from the experiment concerned with the effect of impact velocity was used to parameterize the effect counterweight load on impact velocity. In this experiment, the counterweight was varied while holding the initial angle of the support platform constant. The interested reader is directed to Borrelli et al. [18,25,39] for a detailed discussion of the results from these studies.

In the first experiment, the initial lean angle was varied with no counterweight. Participants performed 8 simulated forward falls. Forward falls were simulated twice from each of 4 initial angles (Low: 65±3, Medium: 52±3, Medium-Hight: 45±3, and High: 39±3 degrees). In the second experiment, the counterweight was varied, and the initial lean angle was held constant. Participants performed 8 simulated forward falls. Two simulated forward falls were performed with each of 4 counterweights (Small: 6.8±0.4, Medium: 12.4±0.8, Medium-Large: 17.9±1.1, Large: 29.1±1.7 %BW) beginning from the same initial angle (37±1 degrees from vertical). Participants were afforded a break, up to 2 minutes, between each trial to minimize fatigue. The initial lean angle (Experiment 1) and counterweight (Experiment 2) were randomly selected for each participant. Prior to starting, participants performed a single familiarization trial using the same initial lean angle or counterweight load that would be used in the first 2 trials.

The starting conditions and instructions were the same in both experiments. Participants began in a leaning position with the ankles plantar flexed and the hands resting on the top of the support platform (Fig. 1A). The starting position necessitated rapid orientation of the hands/arms prior to impact. The shoulders were positioned approximately 12 inches above the top of the support platform. The support platform was released at an unpredictable time. Participants were instructed to “land on their hands as softly as possible.” Each hand landed on a separate gymnastic mat (North Carolina Gym Supply, Hillsborough, NC) with a force platform (AMTI, Watertown, MA) below it.

### Participants

Fourteen younger adults (5 males/9 females; mean age ± standard deviation (sd) (range): 25.1±3.5 years (22-32 years); mean height: 173.9±10.0 cm (159-191 cm); mean body mass: 78.9±4.6 kg (54.4-103.0 kg)), and 13 older adults (9 males/4 females; mean age: 71.3±3.7 years (65-77 years); mean height: 175.2±9.0 cm (157-189 cm); mean body mass: 77.3±15.0 kg (56.3-104.8 kg)) participated in Experiment 1. Thirteen younger adults (12 males/1 female; mean age: 31.8±5.7 years (25-41 years); mean height: 185.7±6.1 cm (174-193 cm); mean body mass: 82.4±5.6 kg (77.4-92.1 kg)) participated in Experiment 2. Experiment 2 ended prematurely because of shut-down due to COVID-19 and older adults were not included. Inclusion and exclusion criteria are described elsewhere [25,39]. Briefly, healthy adults were included and participants were excluded if they currently experienced physical limitations or underwent surgery within 6 months of enrollment. All participants provided written informed consent prior to participation. The study was approved by the Institutional Review Board at the University of Maryland School of Medicine.

### Data recording and processing

The angular velocity of the support platform of the FALL FIT was recorded using a 10 camera Vicon motion capture system with Nexus Software (Oxford, UK). Motion capture data were recorded at 150 Hz and low-pass filtered using a dual-pass second-order Butterworth filter with a cut-off frequency of 10 Hz respectively.

### Model parameter fitting

The angular velocity of the support platform at 70 degrees was used to fit the dynamic model (eqn [13]). Seventy degrees was empirically determined to be the largest angle prior to any participant or the support platform contacting the gym mats. After the gym mat is contacted, the model no longer applies, and the velocity of the table is no longer dependent on only gravity, CoM height, and counterweight load.

**Fig. 7:**
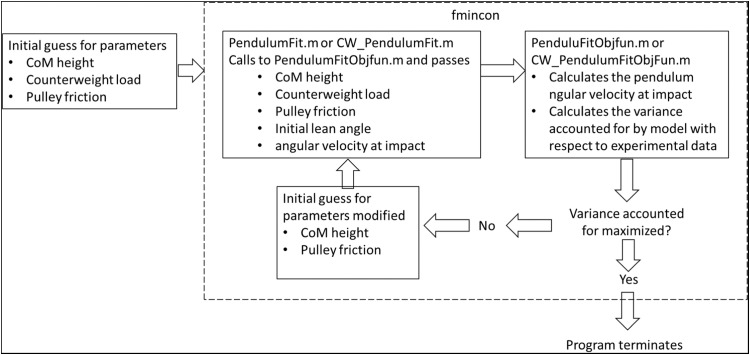
Flowchart representing the process flow for the Matlab scripts written to fit the model parameters to the experimental data. An initial guess for the CoM height, counterweight load (equal to zero for trials with no counterweight) and pulley friction (equal to zero for trials with no counterweight) is provided by the user and used as input to the fmincon function. The fmincon function uses the script PendulumFit.m or CW_PendulumFit.m to calculate the angular velocity at impact using the dynamic model, AtwoodPendulum3.m (eqn [13]). The variance accounted for by the model, with respect to experimental data, is calculated and if it is not maximized, the CoM height and pulley friction are modified and the process repeats until the variance accounted for is maximized.

Custom scripts were written in Matlab using constrained optimization (fmincon in Matlab (Mathworks, Natick, MA)) to fit the model parameters, pendulum length (*L*) in Experiments 1 and pendulum length and drag friction coefficient (*μ_F_*) in Experiment 2, in eqn [14] and eqn [13] respectively. Model parameters were selected such that the model predicted angular velocity maximized the variance accounted for, *r^2^*, when compared to data measured in experiment. The Matlab scripts are included in the Appendix and a flowchart representing the process is shown in Fig. 7.

### Representative Results

**Fig. 8:**
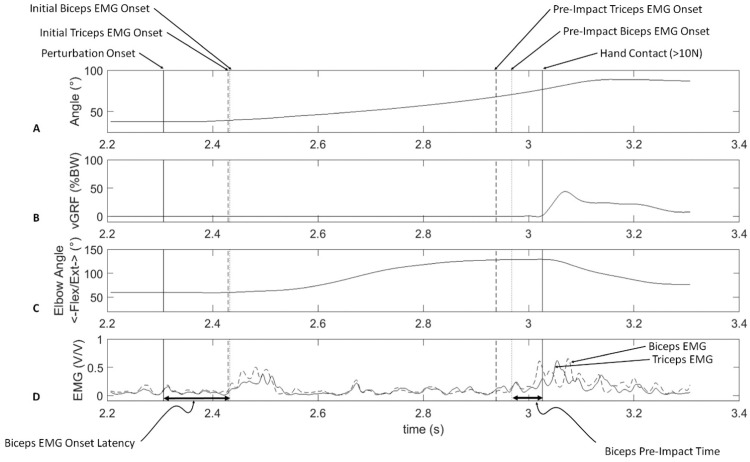
Fig. 2 Example data from a trial with an initial lean angle of 38 degrees from the vertical and counterweight mass of 24.6 kg. The corrected counterweight was 23.1% body weight when weight of the participant and support platform (14.4 kg) are considered the total mass. Panel A shows the angle of the support platform as a function of time. Panel B shows the vertical ground reaction force (GRF) under the preferred hand. Panel C shows the elbow angle. Participants’ elbows were initial flexed with the hands in front of the chest. Following perturbation onset, the elbows are extended until a short time prior to impact. Following impact, the elbows are flexed. The activity of the biceps and triceps are shown in panel D. There are two bursts of EMG activity in the biceps and triceps between perturbation onset and initial impact. The first burst of activity of the biceps and triceps, the EMG onset latency, occurs 145(53) and 132(34) ms after perturbation onset respectively. The burst of biceps and triceps activity preceding impact, the pre-impact time, occurs 98 (58) and 118 (50) ms before impact respectively. From left to right, the vertical lines represent perturbation onset (solid line), initial triceps EMG onset (dashed line), initial biceps EMG onset (dotted line), pre-impact triceps EMG onset (dashed line), pre-impact biceps EMG onset (dotted line), and hand impact with the landing surface (sold line). Adapted from Borrelli, Creath, and Rogers (2023) with permission.

A few studies have been conducted using the FALL FIT. Investigations have been conducted with varying initial lean angles with no counterweight in younger adults [18] and older adults [25], with varying counterweight and constant initial lean angle in younger adults [39], and the reliability of repeated measurements has been established [41]. Fig. 8 shows example data from a single trial with an initial lean angle of 37 degrees from the vertical and a counterweight load of 23.1% bodyweight. At an unpredictable time, the electromagnet is released (perturbation onset) and the support platform rotates until it reaches 90 degrees from the vertical when it contacts the gym mats and shock absorbers (Fig. 8**A**). Initial impact with the landing surface is assumed to occur when the vertical ground reaction force exceeds 10 Newtons (Fig. 8**B**). Following perturbation onset, the elbows are extended rapidly in preparation for impact (Fig. 8**C**). About 120 ms after perturbation onset, a burst of EMG activity in the triceps and biceps is observed followed by another separate burst of activity that occurs in preparation for impact [39] (Fig. 8**D**).

**Fig. 9:**
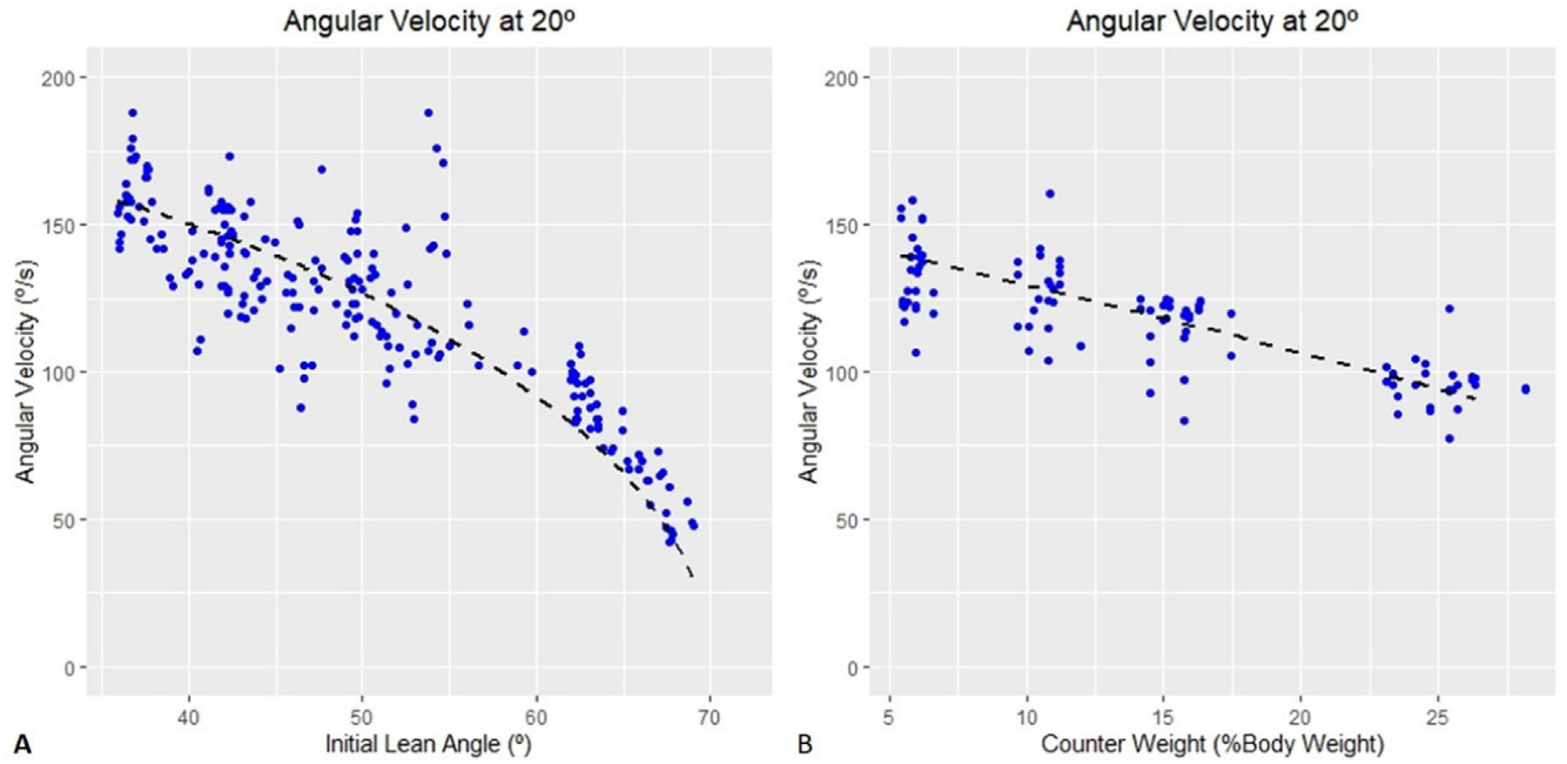
Model predicted angular velocity at various lean angles with no counterweight (panel **A**) and model predicted angular velocity at various counterweights with the initial lean angle held constant at 37 degrees (panel **B**). The initial lean angle account for more than 72% of the variance of the angular velocity of the support platform at 70 degrees in (panel **A**) and the counterweight load accounted for 60% of the variance of the angular velocity of the support platform at 70 degrees in (panel B). The dotted black line is the angular velocity predicted angular velocity predicted by the model and the blue dots are experimental results.

The data from our previous work was used to fit the dynamic model described above. The angular velocity predicted by equation [14] and experimental data from experiments without a counterweight [18,25] are shown in Fig. 9**A**. The pendulum length that maximized the variance accounted for by the initial lean angle was *L*=1.21 m. Initial lean angle predicted 72% of the measured variance in angular velocity at 70 degrees.

The angular velocity predicted by equation [13] and experimental data from experiments with a counterweight [39] are shown in Fig. 9**B**. Fig. 9**B** shows the angular velocity predicted by equation [13] and experimental data from the experiment with varying counterweight. The pendulum length and drag friction that maximized the variance accounted for are 1.30 m and 0.18 [unitless] respectively. The resulting model predicted that counterweight load accounted for 60% of the variance in angular acceleration at 70 degrees.

## Discussion

We have developed a simple method using a system of cables and pulleys to modulate angular velocity in simulated forward falls. We have also developed a dynamic model that predicts the angular velocity of the support platform just before impact. The potential for investigating protective arm reactions has been demonstrated in younger and older adults [25,39,42] and the reliability or repeated measures of the FALL FIT has been established.

An important difference between this method and others used to investigate protective arm reactions [21,[26], [27], [28], [29],^32^,^43^] is the requirement for rapid arm orientation, needed to support body weight after impact, and the ability to provide an unpredictable fall velocity. Generally, fall velocity is a function of fall height (initial angle), the height of the CoM allowing preplanning when the fall height is fixed in advance of an expected perturbation, and the arms are pre-positioned in preparation for impact [21,26,32,42,43]. Alternatively, in studies where rapid arm movement is required, body weight support is not required after impact because participants reach from a seated or standing position [24,28,29]. Future work should investigate the possibility of using a large enough counterweight load such that the fall can be prevented by rapid arm movements and/or counterbalancing allowing an additional layer of realism, i.e., participants must choose to try and recover balance or to use the hands arms to protect the body from injury. Furthermore, this paradigm offers control of impact velocity allowing the impact velocity to be consistent between participants with different heights.

The model predicted that the angular velocity of the support platform accounts for more than 60% of the variance observed experimentally as a function of initial lean angle and counterweight load. Inverted pendulum models of human movement often assume the mass of the pendulum is located at the same height as the CoM. The estimates of pendulum height were 69% height in experiment 1 (*L*=1.21 m) and 60% height in experiment 2 (*L*=1.30 m). The CoM is located at about 55% of an individual's height [44] and the average height of the participants was 1.75 m (0.96 m is 55% of 1.75) and 1.84 m (1.02 m is 55% of 1.84 m) in experiment 1 and experiment 2 respectively. Given that the average foot length is 24 cm [45], standing with the ankles plantarflexed and the arms resting near the height of the the shoulders, participants’ CoM is effectively increased and may explain the discrepancy between CoM location and the estimate from our model. Furthermore, the participants are lying atop 12 inches of foam.

Control of protective arm reactions have been shown to be affected by initial lean angle and coutnerweight load or impact velocity [[18], [25], [39]]. Drop duration, maximum vertical ground reaction force, and vertical neck velocity have been shown to be dependent on initial lean angle and counterweight load. Vertical hand velocity and average EMG amplitude preceding impact were affected by counterweight load but were not affected by initial lean angle. Elbow angle and the timing of the burst of EMG activity in preparation for impact (with respect to impact timing) were affected by initial lean angle but not by counterweight load [[18], [25], [39]]. Control of impact velocity and intial lean angle may facilitate future sutdies in untangling the interplay between these biomechanical factors and fall characteristics which affect the effectiveness of protective arm reactions.

The device itself is relatively inexpensive with materials and fabrication cost less than $5,000 USD. However, the cost of the associated data collection equipment, motion capture, EMG, and force platforms, substantially increase the cost of deploying such a system. Future work should explore the potential use of the FALL FIT as a training and assessment tool in the clinic by relying on observation, patient feedback, and clinical assessment tools. However, the utility of the FALL FIT system in reducing fall injury risk and deployment as a clinical tool remains to be determined.

## Funding

This work was supported by the University of Maryland Claude D Pepper-OAIC NIH/NIA grant P30AG028747, the University of Maryland Advanced Rehabilitation Research Training Program (NIDRR 90AR5028, NIDILRR 90AR5004 formerly H133P100014).

## CRediT authorship contribution statement

**James Borrelli:** Conceptualization, Methodology, Software, Formal analysis, Investigation, Data curation, Writing – original draft, Writing – review & editing, Visualization, Project administration, Funding acquisition. **Robert A. Creath:** Conceptualization, Methodology, Software, Formal analysis, Supervision. **Mark W. Rogers:** Conceptualization, Methodology, Resources, Writing – review & editing, Supervision, Project administration, Funding acquisition.

## Declaration of Competing Interest

The authors declare that they have no known competing financial interests or personal relationships that could have appeared to influence the work reported in this paper.

## Data Availability

Data will be made available on request.

## Appendix Supplementary materials

Image, application 1
