# Supplementary material for: A method for simulating forward falls and controlling impact velocity
Source: MethodsX. 2023 Sep 26;11:102399. doi: 10.1016/j.mex.2023.102399 (PMC10565865; doi:10.1016/j.mex.2023.102399)
Supplement: Supplementary file 1 [file mmc1.docx]

**Supplementary material**

The Matlab scripts PendulumFit.m or CW_PendulumFit.m are used to estimate the pendulum length that maximizes the variation of angular velocity before impact accounted for by initial lean angle (PendulumFit.m) or counterweight load (CW_PendulumFit.m). Each of these scripts call to a function, PendulumFitObjFun.m and CW_PendulumFitObjFun.m respectively. These functions estimate the impact velocity using the dynamic model of the FALL FIT which is contained in a single file, AtwoodPendulum3.m. The estimated impact velocity is compared with the experimentally determined value and *r^2^* is calculated. The fmincon procedure varies the pendulum length to optimize *r^2^*.

Experimental data, saved in a file name “PendulumFitData.xls”, is used as input. Experimental data should be saved with participant number, initial lean angle as a categorical variable, initial lean angle in degrees from horizontal, and the impact velocity in the first 4 columns when using PendulumFit.m. The format of PendulumFitData.xls is different when using CW_PendulumFit.m. In that case, the data should be saved with participant number, initial table angle in degrees, replicate #, counterweight load in kg, participant weight in Newtons, normalized counterweight load, and angular velocity before impact in the first 9 columns. The normalized counterweight load is in percent and defined to be the counterweight load divided by the sum of the participant weight and the weight of the support platform.

**PendulumFit.m**

x0 = [100;1.1;0];

[x,fval]=fmincon('PendulumFitObjfun',x0,[],[],[],[],[100 1.1 0],[100 1.25 0])

r = 1 - fval/1000

**PendulumFitObjfun.m**

function f = PendulumFitObjfun(x)

g = 9.81; %m/s/s

r=0.115; %Support platform pulley radius

%experimentally determined impact velocity

%columns: [participant, InitialTableAngle(Category 1-4),InitialTableAngle (degrees from horizontal), ImpactAngularVelocity (degrees/s)]

data = xlsread('PendulumFitData');

temp = zeros(length(data(:,1)),1);

SStot = sum((data(:,5)-mean(data(:,5))).^2);

for i = 1:length(data(:,1))

m2 = 0;

theta_0 = (90-data(i,4))*pi/180;

[t,y] = ode45(@(t,y) AtwoodPendulum3(t, y, x, m2, r), [0 2], [theta_0 0]);

tq=linspace(0,2,10000);

yq = interp1(t,y,tq,'spline');

temp(i) = [180*yq(find(yq(:,1)>70*pi/180,1),2)/pi];

clear t y

end

SSres = sum((temp+data(:,5)).^2);

r = 1-SSres/SStot

f = 1000.*SSres/SStot;

xlswrite('Predicted.xls',[data(:,4) -temp])

end

**AtwoodPendulum3.m**

function dy = AtwoodPendulum3(t,y,x,m2,r)

g=9.81;

C1 = exp(x(3)*pi)+exp(2*x(3)*pi)+exp(3*x(3)*pi)+exp(4*x(3)*pi);

dy = zeros(2,1);

%Pendulum equation in state space (Eqn [13])

dy(1) = y(2);

dy(2) = (g*x(1)*x(2)*sin(y(1)) - C1*g*m2*r)/(x(1)*x(2)^2 + 4*C1*m2*r*r);

end

Files to fit parameters for the counterweight system

**CW_PendulumFit.m**

x0 = [100;1.2874;0.1780]; %input is x0 = [system mass, CoM height, friction]

[x,fval]=fmincon('CW_PendulumFitObjfun',x0,[],[],[],[],[100 0.75 0],[100 1.3 0.3])

r_squared = 1 - fval/1000

**CW_PendulumFitObjFun.m**

function f = PendulumFitObjfun(x)

g = 9.81; %m/s/s

theta_0 = 37*pi/180; % 52 degrees from horizontal, initial angle

r=0.115;

%experimentally determined impact velocity

%columns: [participant #, Initial Table Angle (in degrees), Replicate #(1or2), CounterWeight (kg), Participant Weight (N), CounterWeight (kg), Participant Weight (N),EffectiveCounterWeight (%, CounterWeight Load/(ParticipantWeight + SupportPlatformWeight), ImpactAngularVelocity (degree/s)]

data = xlsread('CW_PendulumFitData_2');

temp = zeros(length(data(:,1)),1);

%Total sum of squares for experimental data

SStot = sum((data(:,9)-mean(data(:,9))).^2);

for i = 1:length(data(:,8))

m2 = data(i,8);

[t,y] = ode45(@(t,y) AtwoodPendulum3(t, y, x, m2, r), [0 2], [theta_0 0]);

tq=linspace(0,2,10000);

yq = interp1(t,y,tq,'spline');

%angular velocity at impact

temp(i) = [180*yq(find(yq(:,1)>70*pi/180,1),2)/pi];

clear t y

end

%residual sum of squares

SSres = sum((temp-data(:,9)).^2);

r = 1-SSres/SStot

f = 1000.*SSres/SStot;

xlswrite('CW_Predicted.xls',[data(:,8) -temp])

end
